# A comparison of the effects of fire on rodent abundance and diversity in the Great Basin and Mojave Deserts

**DOI:** 10.1371/journal.pone.0187740

**Published:** 2017-11-28

**Authors:** Tiffanny R. Sharp Bowman, Brock R. McMillan, Samuel B. St. Clair

**Affiliations:** Department of Plant and Wildlife Sciences, Brigham Young University, Provo, Utah, United States of America; University of Missouri Kansas City, UNITED STATES

## Abstract

As invasive grasses and fire increase in frequency and extent in North American deserts, they have the potential to affect animal communities through bottom-up forces. We experimentally tested the effects of fire on rodent communities of the Great Basin and Mojave Deserts. Fire decreased the abundance, richness, and diversity of rodents in the Great Basin after fire. In the Mojave, abundance was unaffected and diversity and species richness were greater on burned than unburned plots 4 months after fire. The effects of fire on rodent communities tended to decrease over time. The differences in effects between the deserts may be due to differences in the foraging preferences of the dominant species at each site. As these species are primarily herbivorous, short-term changes to the rodent community could have long-term implications by affecting the recovery of the plant community after fire.

## Introduction

The invasion of exotic grasses, particularly cheatgrass (*Bromus tectorum*) and red brome (*B*. *rubens*), in North American deserts has dramatically increased the size and frequency of wildfire in these ecosystems [1]. These invasive plant species fill plant interspaces with fine fuels that allow wildfire to carry across large areas. This exotic vegetation recovers quickly after fire and matures and dies early in the season, which can extend the fire season and has increased fire frequency from century to decadal time scales [2–4]. Altered fire regimes and subsequent changes to the plant community can impact animal communities [5].

The responses of different rodent species to fire vary in desert ecosystems. Typically, bipedal species (e.g. kangaroo rats) forage in open areas between shrubs in unburned habitat [6,7] and maintain or increase their abundance when shrub cover is reduced by fire [8–11]. In contrast, quadrupedal species (e.g. mice) often focus foraging efforts under and near shrubs in desert habitat [6,7,12] and decrease in abundance after fire [8,9,11,13,14]. These species-specific responses to altered fire regimes can alter the composition and behavior of rodent communities [15]. While direct mortality of rodents due to fire is rare, indirect impacts due to habitat changes can alter the richness, abundance, and diversity of rodent communities [16–19]. There is mixed evidence that species richness, diversity, or overall abundance of rodent communities in deserts is sometimes greater on unburned than burned areas [11,13,14,20–22], although sometimes no difference is detected in one or more of these measures [9,10,14,21,23]. Despite the fact that rodent responses to fire have been the focus of many studies, the overall results are inconclusive. In addition, the time frame of many previous studies has been at least a year after fire occurred and typically only include one or a few time points [9,11,13,14]. Little is known about the short-term and time dependent responses of rodent communities to fire. Information collected before fire and after fire is vital to understanding how and when changes in richness, abundance, and diversity occur. Furthermore, few studies have compared rodent community responses to fire in different desert ecosystems.

Rodents are keystone species in western North American deserts [24–26], therefore changes to their richness, abundance, or diversity can have important biological feedbacks on plant community characteristics [15]. Rodents affect plant diversity and structure via folivory, granivory, and soil disturbance [24,26]. Because rodent responses to fire vary by species [10], fire can change the diversity and dynamics of the rodent community thus impacting the plant community and possibly the way it recovers after fire [15]. An improved understanding of the changes to the rodent community soon after fire could inform our understanding of the post-fire re-establishment of the plant community and ultimately the changes occurring after fire across desert ecosystems.

The objective of this study was to examine the effects of fire on rodent habitat use in desert ecosystems at small burn scales. We designed an experiment utilizing mark-recapture methods to test the short-term effect of fire on rodent communities in two North American deserts. We hypothesized that fire would impact the abundance, richness, and diversity of the rodent community by limiting resource availability and altering habitat structure. Specifically, we predicted that: i) the abundance of bipedal species (kangaroo rats) would be greater in burned than unburned plots or remain unchanged; ii) the abundance of quadrupedal species (e.g. deer mice, pocket mice) would be lower in burned than unburned plots; iii) rodent species richness and diversity would decrease on burned plots; iv) fewer changes to the rodent community would be observed after fire at a site dominated by a bipedal species than at a site dominated by a quadrupedal species.

## Methods

The research was approved by the Brigham Young University IACUC committee under the following permit number: 120202. The BLM and Brigham Young University’s Lytle Ranch Preserve provided access to study sites.

### Study sites

The study sites were located in the Great Basin and Mojave Deserts in Utah, United States of America. The Great Basin site was located in a sage-steppe community on Bureau of Land Management (BLM) land in Rush Valley (40°5’21.18”N, 112°18’26.88”W). Dominant vegetation was Wyoming sagebrush (*Artemisia tridentata wyomingensis*) and bottlebrush squirreltail (*Elymus elymoides*). The Mojave study area was located at Lytle Ranch Preserve in the Beaver Dam Wash region of southwestern Utah in mid elevation Mojave shrubland (37°8’53.46”N, 114°0’49.59”W). The dominant vegetation at this site was composed of Joshua tree (*Yucca brevifolia*), blackbrush (*Coleogyne ramosissima*), and creosote bush (*Larrea tridentata*). These study sites were established in 2011.

### Plot design

This experiment is part of a larger study looking at the effects of rodents and fire on plant community characteristics. At both study sites (Mojave, Great Basin) the experimental design is identical with five replicated blocks quartered into four adjacent square plots (30 m x 30 m). Adjacent blocks were approximately 50 m apart. Each plot was fenced with welded wire fencing that extended 65 cm aboveground and 35 cm belowground to regulate rodent access. The four plots were randomly assigned within each block to one of four treatments: burned-rodent exclusion, burned-rodent access, unburned-rodent exclusion, and unburned-rodent access. To facilitate movement of rodents into rodent access plots a 15 cm x 15 cm hole was cut into the welded wire at ground level every 3 meters along the outside fence line. Rodents were frequently observed moving between plots and occasionally observed moving between blocks. In this paper, we only analyze and present data for burned and unburned, rodent access plots that allowed rodents to move freely in and out of the plots. Burn treatments were conducted on June 18 (Mojave) and September 17 (Great Basin) of 2011. Burn coverage and severity was high, removing more than 95% of vegetation in all plots.

In the Mojave plots, there was sufficient continuous vegetation to carry fire across the plots. In the Great Basin plots, large shrub interspaces made it hard for fire to carry. Wheat straw was used to carry fire through the plots according to the methods outlined in [15].

### Rodent trapping

We sampled rodents by live trapping within each plot and outside of each plot before and after controlled burns occurred. The Mojave site was sampled 3 weeks prior to the burn and 3 and 17 weeks after the burn; subsequent sampling occurred three times annually (spring, summer, and fall) from 2012 to 2014. We attempted to sample the Great Basin site along the same timeline; however, due to burn restrictions the date of the fire was postponed. This site was therefore sampled 12 and 9 weeks prior to the burn and 3 weeks afterwards; as with the Mojave site, subsequent sampling occurred three times annually through 2014. For each trapping session eight Sherman live traps were placed 1 m inside the fencing of each plot, spaced 10 m from the nearest trap and 10 m from the plot edges with two traps per side. Four traps were placed 10 m outside the fencing of each plot that served as controls. A total of 240 traps were set each night at each study area (S1 Fig). Traps were set each evening and checked each morning for three consecutive nights. Each trap was baited with commercially available rodent seed mix. Polyester batting was added to traps if temperatures were predicted to drop below 4°C to reduce the likelihood of exposure. Rodents received an individually numbered ear tag and the species, trap location, sex, age, reproductive condition, and mass were recorded for each animal. Because tags are occasionally lost from pocket mice with small ears [27], we also shaved a small patch of fur from these animals when captured to identify them as recaptures during subsequent nights within the capture session. We rarely saw ear tags lost from the other rodent species. All animals were released at the point of capture. All capture and handling methods were approved by the Institutional Animal Care and Use Committee (IACUC) of Brigham Young University (Protocol Numbers 090302 and 120202).

### Data analysis

Analysis of variance tests were used to examine burn effects. All analyses were performed with program R Version 3.1.1 [28]. The number of unique individuals recorded in each time period was used as our measure of rodent abundance. This metric is biased low and is robust to variation in capture probability [29]. Species richness (number of species), overall abundance (total number of individuals in each species), reciprocal Simpson diversity index, and Shannon diversity index (indices that take into account number of species and relative abundance of each species) were calculated for each plot during each time period using the function diversityresult in the BiodiversityR package. These four values as well as species-specific abundance were used as response variables for analyses. To determine if there were short-term effects of fire on rodents, we tested for a treatment by time interaction with a separate model for each response variable using function lmer in package lme4. Separate models were run for each desert. These models took the form: response ~ treatment + time + treatment*time + (1|block). Treatment was a factor with two levels (burned or unburned), time a factor with ten levels (one for each trapping period), and block a factor with five levels included as a random effect to account for spatial effects. When ANOVA tests were significant, Tukey adjusted pairwise comparisons of least squared means were conducted using function lsmeans in package lsmeans [30]. These tests were used to determine if there were differences between unburned and burned plots in each time period. Data exploration was conducted according to the methods of Zuur et al. [31] to test that all model assumptions were met including normality and homogeneity of variance. Residuals for each model were checked and assumptions were only violated for species with few numbers of captures. These were subsequently excluded from the analysis.

## Results

We had 1,018 captures of 487 individual rodents in the Great Basin and 1,244 captures of 505 individuals in the Mojave over 12,960 total trap-nights. In decreasing order of abundance, species comprising the community in the Great Basin were deer mouse (*Peromyscus maniculatus*), chisel-toothed kangaroo rat (*Dipodomys microps*), Great Basin pocket mouse (*Perognathus parvus*), least chipmunk (*Tamias minimus*), sagebrush vole (*Lemmiscus curtatus*), and northern grasshopper mouse (*Onychomys leucogaster*). In decreasing order of abundance, species comprising the community in the Mojave were Merriam’s kangaroo rat (*D*. *merriami*), long-tailed pocket mouse (*Chaetodipus formosus*), desert woodrat (*Neotoma lepida*), canyon mouse (*Peromyscus crinitus*), northern grasshopper mouse, white-tailed antelope squirrel (*Ammospermophilus leucurus*), and desert cottontail (*Sylvilagus audubonii*).

In the Great Basin, we captured four species of rodent in burned plots, five in unburned plots, and five outside of the plots. Chisel-toothed kangaroo rats, deer mice, Great Basin pocket mice, and least chipmunk were captured in burned plots. All of these species were captured in unburned plots and outside plots with the addition of the sagebrush vole in unburned plots and the northern grasshopper mouse outside the plots. We did not perform species-specific abundance comparisons for any species with less than twenty total unique individuals recorded throughout the study; in the Great Basin these species were the sagebrush vole and northern grasshopper mouse. Great Basin rodent abundance (t = 0.00, p = 1.00), diversity (Simpson’s: t = 0.49, p = 0.87; Shannon’s: t = 0.82, p = 0.69), and species richness (t = 0.88, p = 0.65) did not differ between unburned plots and areas outside the experimental plots giving us confidence that our plots accurately reflected the natural use patterns of rodents in our study system. Total abundance (t = 0.88, p = 0.65), diversity (Simpson’s: t = 0.73, p = 0.75; Shannon’s: t = 1.23, p = 0.44), and species richness (t = 0.90, p = 0.64) also did not differ between burned and unburned plots before the burn treatment was applied in June 2011.

In the Mojave, we captured six species of rodent in burned plots, seven in unburned plots, and seven outside of the plots. Merriam’s kangaroo rats, long-tailed pocket mice, desert woodrats, canyon mice, northern grasshopper mice, and white-tailed antelope squirrels were captured in burned plots. All of these species and desert cottontails were captured within unburned plots and outside of the plots. We did not perform species-specific abundance comparisons for any species with less than twenty total unique individuals recorded; in the Mojave these species were the canyon mouse, northern grasshopper mouse, white-tailed antelope squirrel, and desert cottontail. Mojave rodent abundance (t = 0.59, p = 0.82), diversity (Simpson’s: t = 1.23, p = 0.44; Shannon’s: t = 1.08, p = 0.53), and species richness (t = 0.75, p = 0.74) did not differ between unburned plots and areas outside the experimental plots. Total abundance (t = 0.14, p = 0.99), diversity (Simpson’s: t = 0.01, p = 1.00; Shannon’s: t = 0.58, p = 0.83), and species richness (t = 0.37, p = 0.93) also did not differ between burned and unburned plots before the burn treatment was applied in May 2011.

### Effects of fire on rodents

**Fig 1:**
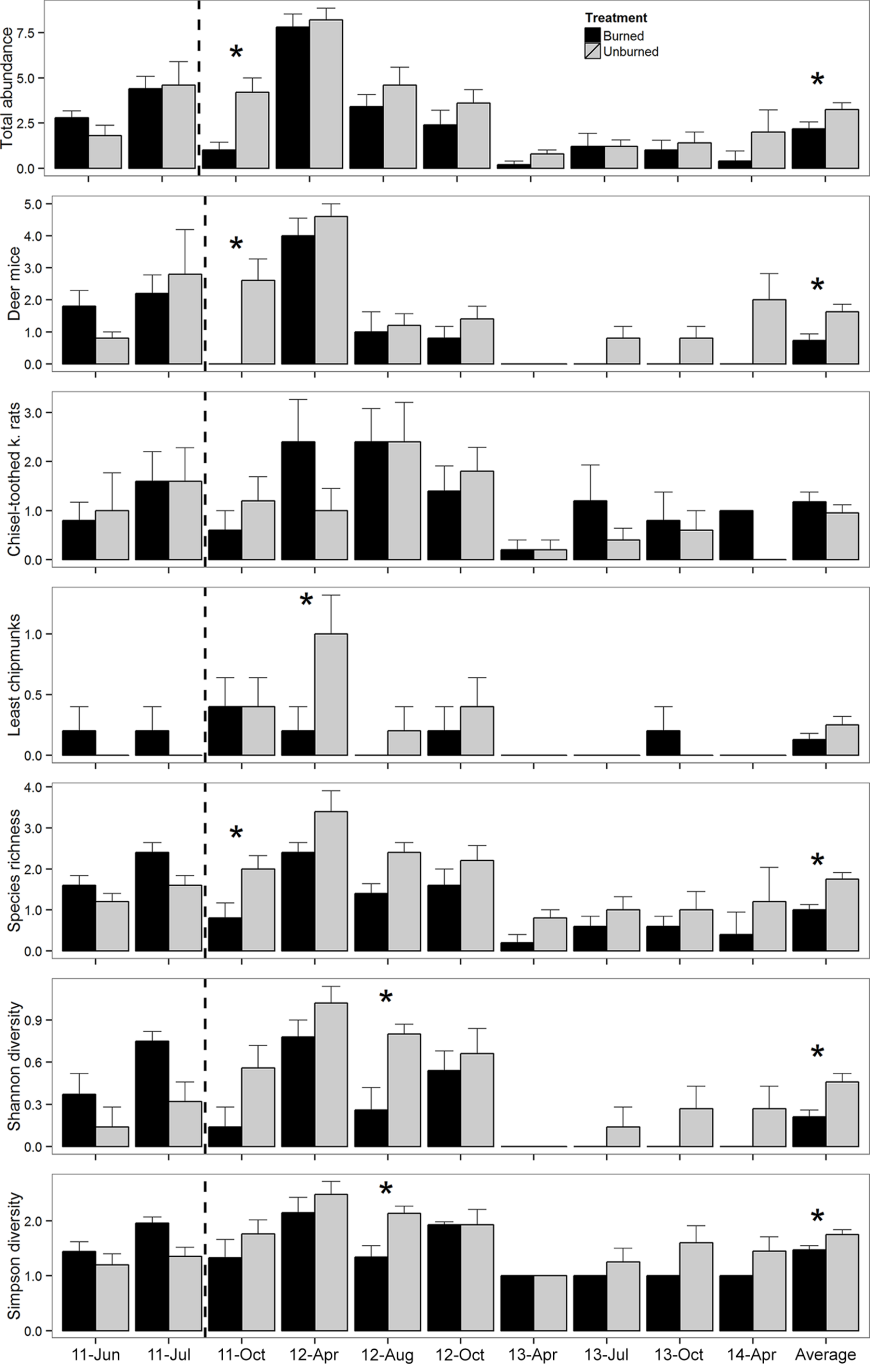
Abundance of all rodent species, abundance of deer mice, abundance of chisel-toothed kangaroo rats, abundance of least chipmunks, species richness, Shannon and Simpson diversity indices in burned and unburned plots (+SE) in the Great Basin desert between June 2011 and April 2014. The dashed line marks the time when plots were burned; * denotes significant difference (p<0.05) between burned and unburned plots for a given trapping occasion.

In the Great Basin, rodent abundance, richness, and diversity decreased in burned plots after fire. In October 2011, three weeks after fire, total abundance (burned: 1.0 ± 0.4; unburned: 4.2 ± 0.8; t = 2.84, p = 0.02), deer mouse abundance (burned: 0 ± 0; unburned: 2.6 ± 0.7; t = 2.90, p = 0.01), and species richness (burned: 0.8 ± 0.4; unburned: 2.0 ± 0.3; t = 2.70, p = 0.02) were lower in burned than unburned plots (Fig 1). In April 2012, seven months after the fire, least chipmunks were less abundant in burned (1.0 ± 0.3) than unburned plots (0.2 ± 0.2; t = 2.50, p = 0.04); abundance of least chipmunk did not differ in any other time period (all p>0.10). In August 2012, 11 months after fire, species diversity was lower in burned than unburned plots for both the inverse Simpson (burned: 1.34 ± 0.21; unburned: 2.14 ± 0.13; t = 2.43, p = 0.04) and Shannon (burned: 0.26 ± 0.16; unburned: 0.80 ± 0.07; t = 2.90, p = 0.01) indices. Abundances of chisel-toothed kangaroo rat and Great Basin pocket mouse did not differ between burned and unburned plots in any time period (all p>0.10). Average post-fire deer mouse abundance (burned: 0.7 ± 0.2; unburned: 1.6 ± 02; t = 4.04, p<0.01), overall abundance (burned: 2.2 ± 0.4; unburned: 3.3 ± 0.4; t = 3.33, p<0.01), species richness (burned: 1.0 ± 0.1; unburned: 1.8 ± 0.2; t = 4.30, p<0.01), inverse Simpson diversity (burned: 1.47 ± 0.08; unburned: 175 ± 0.09; t = 3.96, p<0.01) and Shannon diversity (burned: 0.21 ± 0.05; unburned: 0.46 ± 0.06; t = 3.85, p<0.01) were lower in burned than unburned plots.

**Fig 2:**
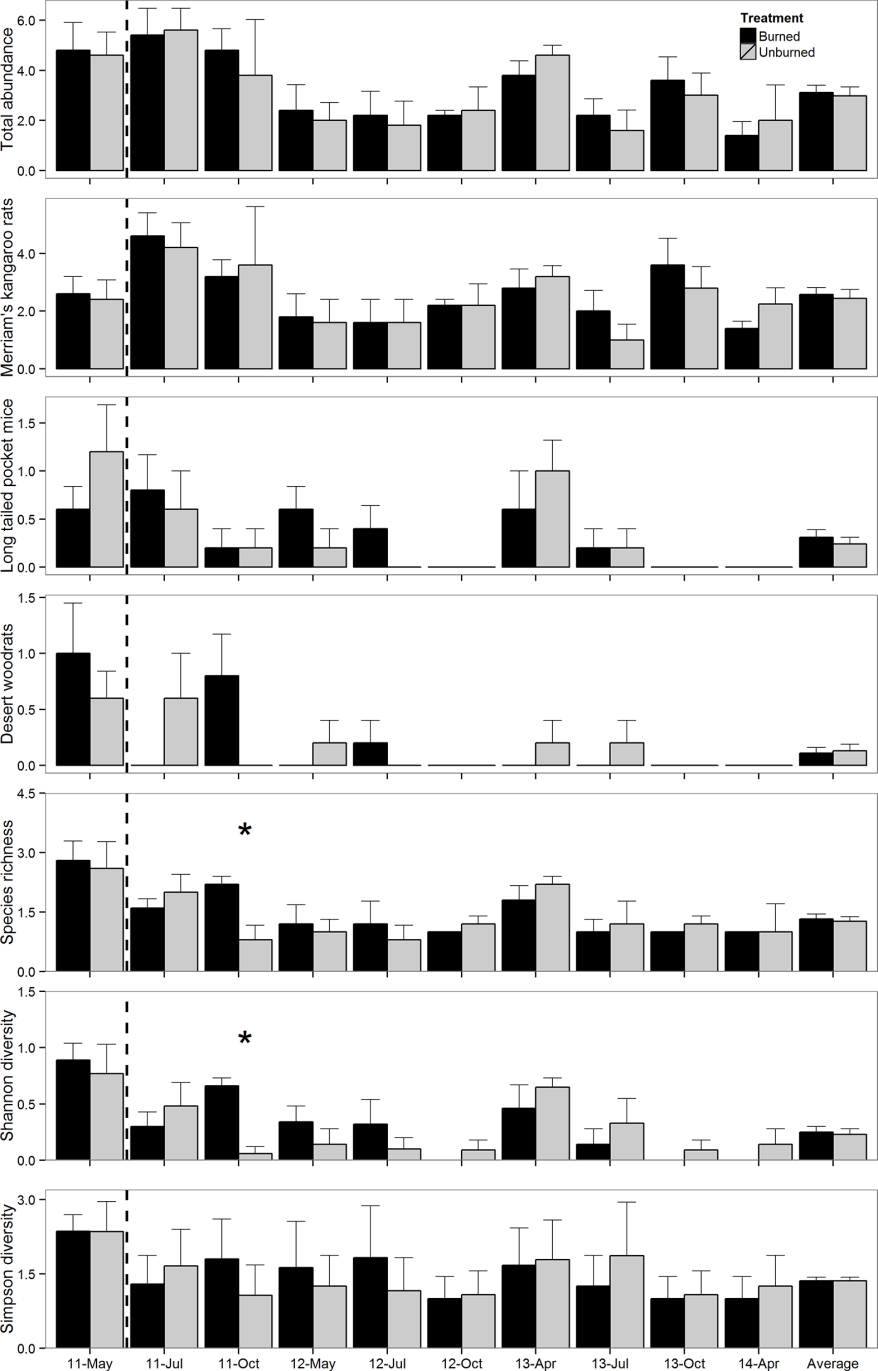
Overall abundance of rodents (+SE), abundance of Merriam’s kangaroo rats, abundance of long-tailed pocket mice, abundance of desert woodrats, species richness, Shannon’s diversity index, and Simpson’s diversity index in burned and unburned plots in the Mojave desert 2011–2014. The dashed line marks the time when plots were burned. Differences (p<0.05) between burned and unburned plots within a trapping occasion are marked with an asterisk (*).

In the Mojave, rodent diversity and species richness increased in burned plots after fire. In October 2011, four months after fire, species richness (burned: 2.2 ± 0.2; unburned: 0.8 ± 0.4; t = 2.59, p = 0.03) and Shannon’s diversity index (burned: 0.66 ± 0.07; unburned: 0.06 ± 0.06; t = 2.90, p = 0.01; Fig 2) were greater in burned than unburned plots; these measures did not differ in any other time period (all p>0.10). Simpson’s diversity index, overall abundance, and all species-specific abundances did not differ between burned and unburned plots in any time period (all p>0.10).

### Comparison across deserts

We observed more differences in the rodent community between burned and unburned plots in the Great Basin than the Mojave. Average species richness, diversity and abundance of deer mice and all species combined were lower in burned than unburned plots in the Great Basin after fire. However, no differences were detected in abundance between burned and unburned plots in any time period before fire or up to 34 months after fire in the Mojave. Species richness and Shannon’s diversity index were greater in burned than unburned plots four months after fire, but no other differences were observed between treatments in the Mojave. Rodent abundance, richness and diversity in both the Great Basin and Mojave Deserts shifted strongly over the period of the experiment (Time effects for the models were all significant: P < 0.0001). In the Great Basin Desert, rodent abundance, richness and diversity was significantly higher from June 2011 through October 2012 than April 2013 to April 2014 (Fig 1). In the Mojave Desert, rodent abundance, richness and diversity tended to be higher during some sampling points in 2011 and 2013 than 2012 and 2014 (Fig 2).

## Discussion

### Great Basin fire effects

Direct fire mortality of rodents tends to be low in forest [32–34], prairie [18], and arid ecosystems [35,36]. The indirect effects of fire mediated by changes in the plant community likely had a much stronger impact on rodent communities [15]. In the Great Basin, there were short-term effects of fire on the abundance of the rodent community. Average abundances of deer mice and all species combined were lower in burned plots than unburned plots after fire. The decline in overall abundance appears to be driven primarily by the change in deer mouse numbers. Lower abundance of deer mice at burned than unburned sites has previously been demonstrated in desert habitats [13]; however, it is interesting to note that this species often exhibits a positive response to fire in forest and prairie habitats [18,37–40]. Differences in litter cover and interspecific competitors may be responsible for different fire responses in different habitats [18,37,38].

In the Great Basin, the effects of fire on the rodent community were strong early on and significant when averaged across time. However, significant fire effects at individual time points were not detected 1–2.5 years after fire. Likewise, previous research in the Great Basin found that diversity did not differ 6–17 years after fire [14]. However, greater abundance and richness were found in unburned than burned habitat 1–17 years after fire [13,14].

### Mojave fire effects

In the Mojave, there were no effects of fire on the abundance of the rodent community and few effects on richness and diversity. Species richness and Shannon’s diversity index were both greater in burned than unburned plots four months after fire; afterwards, no differences were detected in these measures. However, our results are inconsistent with those of a study performed on naturally occurring burned and unburned habitats near our Mojave study plots [11]. At these nearby sites, the abundance of Merriam’s kangaroo rat increased, while the abundances of long-tailed pocket mouse, canyon mouse, and all species combined decreased on sites burned 4–5 years previously compared to unburned sites. That study also found reduced richness and diversity at burned sites. These changes in abundance, richness, and diversity may be the result of accumulated indirect effects of fire impacting the survival or reproductive rates of rodents in burned areas over time. Similar changes to the rodent community may be occurring on our site, yet they remain undetected at this relatively early time.

The fire effects that we detected on the rodent community in the Mojave were all within four months of the burn; no differences were detected ten months to three years after fire. In contrast, previous studies have found that diversity has been greater in unburned than burned Mojave habitat two or more years after fire [11,21]. There is mixed evidence as to whether abundance and species richness are greater in unburned habitat [11] or do not differ [21] as in our study.

### Desert comparisons

We hypothesized that the mode of locomotion of the most abundant species would influence how each community responded to fire. At our Great Basin site deer mice are the most abundant species and are quadrupedal; quadrupedal species tend to prefer shrub cover [6,7,12] which is decreased by fire. At our Mojave site, Merriam’s kangaroo rats are the most abundant and are bipedal; bipedal species tend to prefer open areas between shrubs which are increased by fire [6,7]. We therefore expected more severe decreases in the abundance, richness, and diversity of rodents in the Great Basin as their preferred habitat decreased after fire. Consistent with our prediction, we observed more impacts of fire on the rodent community in the Great Basin than in the Mojave (Figs 1 and 2). Similarly, other studies have found decreases in the abundance of quadrupedal species after fire [8,9,11,13,14] and equal or increased abundances for bipedal species after fire [8–11].

As rodents play a keystone role in North American deserts [24–26], understanding their responses to fire in these ecosystems is an important step to understanding the regrowth of the plant community and planning effective rehabilitation efforts for burned regions. Additionally, understanding the longer-term changes to the rodent community through continued monitoring of burned regions will aid in the understanding of how these communities change over time. As these communities shift, the impact on the plant community will have an important influence on these desert landscapes and help to determine how they respond to fire and other disturbances.

Impacts of fire were detected in both the Great Basin and Mojave Deserts within a year after fire. These effects of fire were more numerous and persistent in the Great Basin than the Mojave and this difference may be due to the modes of behavior of the dominant species at each site. These results add to our understanding of the changes occurring in the deserts of western North America as a result of alterations to the fire regime. This information can help us understand the post-fire dynamics of these ecosystems as a whole and inform management decisions regarding post-fire wildland rehabilitation efforts. In addition, rodent populations fluctuated strongly across seasons and years as has been noted in other studies in the Mojave and Great Basin Deserts (11,15). These fluctuations can be related to reproductive or disease cycles in rodents and climatic factors [41]. In our study systems 2012 was a particularly dry year which may have contributed to reductions in the hyper-arid Mojave Desert in 2012 (11). The 2012 drought may have had more of a lag effect in the more mesic and cooler Great Basin Desert (15).

The future of the Great Basin and Mojave Deserts is threatened by invasive grass and associated fire [1,2,4]. By improving our understanding of these systems, we can implement effective management actions that will minimize the impacts of invasive species and preserve these unique ecosystems. Desert rodents, as both plant consumers and dispersers, play an important role in determining the structure of the plant community [24–26] and often plan a critical role in plant invasion outcomes (15). Understanding how rodents are effected by fire, and how that impacts their top-down effects on native plant community assembly and the establishment and spread of invasive species, is pivotal to understanding how these systems respond to invasive grass fire cycles in the Great Basin and Mojave Deserts [42, 43].

## Supporting information

S1 Fig Trap layout in each block; each triangle represents a trap station with one Sherman live trap. Each block contains an adjacent burned and unburned plot (30 x 30 m each) with 8 traps inside each plot and 4 traps outside each plot as plot controls.
(TIFF)

S1 File Minimal data set. (DOCX)
