# Supplementary figures and images for: A comparison of the effects of fire on rodent abundance and diversity in the Great Basin and Mojave Deserts
Source: PLoS One. 2017 Nov 28;12(11):e0187740. doi: 10.1371/journal.pone.0187740 (PMC5705133; doi:10.1371/journal.pone.0187740)

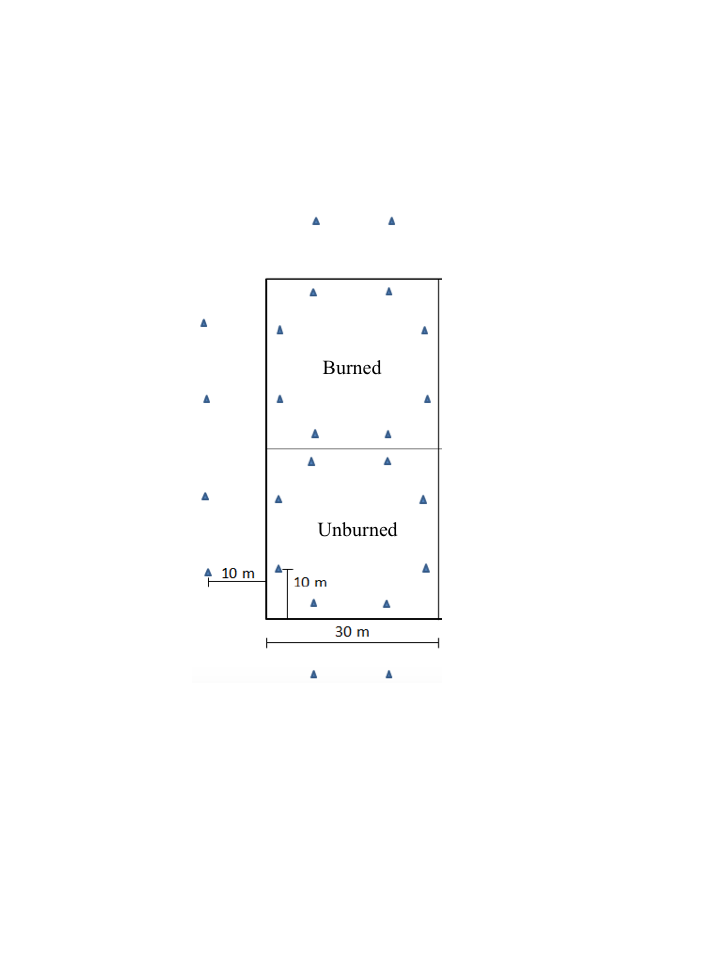

Supplement: S1 Fig — Each block contains an adjacent burned and unburned plot (30 x 30 m each) with 8 traps inside each plot and 4 traps outside each plot as plot controls. (TIFF) [file pone.0187740.s001.tiff]
