# Supplementary material for: A comparison of the effects of fire on rodent abundance and diversity in the Great Basin and Mojave Deserts
Source: PLoS One. 2017 Nov 28;12(11):e0187740. doi: 10.1371/journal.pone.0187740 (PMC5705133; doi:10.1371/journal.pone.0187740)
Supplement: S1 File — (DOCX) [file pone.0187740.s002.docx]

Species-specific abundance data for Great Basin plots. This is the raw data used in R to generate total abundance, species richness, and diversity indices for each plot in each time period. Treatment is represented by B=burned, U=unburned, and O=outside the plots.

| Monthyear.block.treatment | *Dipodomys microps* | *Perognathus parvus* | *Lemmiscus curtatus* | *Peromyscus maniculatus* | *Onychomys leucogaster* | *Tamius minimus* |
| --- | --- | --- | --- | --- | --- | --- |
| 0611.1.B | 0 | 0 | 0 | 3 | 0 | 1 |
| 0611.1.U | 0 | 0 | 0 | 1 | 0 | 0 |
| 0611.1.O | 1 | 0 | 0 | 3 | 0 | 0 |
| 0611.2.B | 0 | 0 | 0 | 2 | 0 | 0 |
| 0611.2.U | 0 | 0 | 0 | 1 | 0 | 0 |
| 0611.2.O | 1 | 0 | 0 | 6 | 0 | 0 |
| 0611.3.B | 2 | 0 | 0 | 0 | 0 | 0 |
| 0611.3.U | 1 | 0 | 0 | 1 | 0 | 0 |
| 0611.3.O | 1 | 0 | 0 | 0 | 0 | 0 |
| 0611.4.B | 1 | 0 | 0 | 2 | 0 | 0 |
| 0611.4.U | 4 | 0 | 0 | 0 | 0 | 0 |
| 0611.4.O | 2 | 0 | 0 | 3 | 1 | 0 |
| 0611.5.B | 1 | 0 | 0 | 2 | 0 | 0 |
| 0611.5.U | 0 | 0 | 0 | 1 | 0 | 0 |
| 0611.5.O | 1 | 0 | 0 | 2 | 0 | 0 |
| 0711.1.B | 3 | 0 | 0 | 2 | 0 | 0 |
| 0711.1.U | 1 | 0 | 0 | 2 | 0 | 0 |
| 0711.1.O | 1 | 3 | 0 | 9 | 0 | 0 |
| 0711.2.B | 1 | 0 | 0 | 4 | 0 | 1 |
| 0711.2.U | 2 | 0 | 0 | 5 | 0 | 0 |
| 0711.2.O | 6 | 3 | 0 | 5 | 0 | 1 |
| 0711.3.B | 3 | 0 | 0 | 1 | 0 | 0 |
| 0711.3.U | 1 | 0 | 0 | 0 | 0 | 0 |
| 0711.3.O | 1 | 3 | 0 | 5 | 0 | 1 |
| 0711.4.B | 0 | 1 | 0 | 1 | 0 | 0 |
| 0711.4.U | 4 | 0 | 0 | 0 | 0 | 0 |
| 0711.4.O | 2 | 1 | 0 | 3 | 0 | 2 |
| 0711.5.B | 1 | 1 | 0 | 3 | 0 | 0 |
| 0711.5.U | 0 | 1 | 0 | 7 | 0 | 0 |
| 0711.5.O | 2 | 2 | 0 | 10 | 0 | 1 |
| 1111.1.B | 2 | 0 | 0 | 0 | 0 | 0 |
| 1111.1.U | 3 | 0 | 0 | 3 | 0 | 1 |
| 1111.1.O | 2 | 0 | 0 | 5 | 0 | 3 |
| 1111.2.B | 0 | 0 | 0 | 0 | 0 | 0 |
| 1111.2.U | 1 | 0 | 0 | 3 | 0 | 0 |
| 1111.2.O | 0 | 0 | 0 | 8 | 0 | 6 |
| 1111.3.B | 1 | 0 | 0 | 0 | 0 | 1 |
| 1111.3.U | 1 | 0 | 0 | 0 | 0 | 1 |
| 1111.3.O | 3 | 0 | 0 | 6 | 0 | 4 |
| 1111.4.B | 0 | 0 | 0 | 0 | 0 | 0 |
| 1111.4.U | 1 | 0 | 0 | 3 | 0 | 0 |
| 1111.4.O | 1 | 0 | 0 | 3 | 0 | 3 |
| 1111.5.B | 0 | 0 | 0 | 0 | 0 | 1 |
| 1111.5.U | 0 | 0 | 0 | 4 | 0 | 0 |
| 1111.5.O | 1 | 0 | 0 | 6 | 0 | 4 |
| 0412.1.B | 3 | 0 | 0 | 4 | 0 | 0 |
| 0412.1.U | 0 | 1 | 0 | 4 | 0 | 1 |
| 0412.1.O | 2 | 2 | 0 | 13 | 0 | 1 |
| 0412.2.B | 3 | 4 | 0 | 3 | 0 | 0 |
| 0412.2.U | 2 | 0 | 0 | 4 | 0 | 2 |
| 0412.2.O | 0 | 3 | 0 | 10 | 0 | 1 |
| 0412.3.B | 5 | 0 | 0 | 4 | 0 | 0 |
| 0412.3.U | 2 | 1 | 0 | 5 | 0 | 1 |
| 0412.3.O | 4 | 2 | 0 | 11 | 0 | 1 |
| 0412.4.B | 1 | 0 | 0 | 6 | 0 | 0 |
| 0412.4.U | 1 | 1 | 1 | 4 | 0 | 1 |
| 0412.4.O | 3 | 2 | 0 | 11 | 0 | 0 |
| 0412.5.B | 0 | 2 | 0 | 3 | 0 | 1 |
| 0412.5.U | 0 | 4 | 0 | 6 | 0 | 0 |
| 0412.5.O | 1 | 4 | 0 | 5 | 0 | 1 |
| 0812.1.B | 5 | 0 | 0 | 0 | 0 | 0 |
| 0812.1.U | 2 | 1 | 0 | 1 | 0 | 0 |
| 0812.1.O | 6 | 1 | 0 | 0 | 0 | 2 |
| 0812.2.B | 2 | 0 | 0 | 0 | 0 | 0 |
| 0812.2.U | 5 | 1 | 0 | 2 | 0 | 0 |
| 0812.2.O | 5 | 0 | 0 | 1 | 0 | 0 |
| 0812.3.B | 2 | 0 | 0 | 3 | 0 | 0 |
| 0812.3.U | 2 | 2 | 0 | 0 | 0 | 0 |
| 0812.3.O | 5 | 0 | 0 | 3 | 0 | 2 |
| 0812.4.B | 2 | 0 | 0 | 0 | 0 | 0 |
| 0812.4.U | 3 | 0 | 0 | 2 | 0 | 0 |
| 0812.4.O | 6 | 3 | 0 | 2 | 0 | 1 |
| 0812.5.B | 1 | 0 | 0 | 2 | 0 | 0 |
| 0812.5.U | 0 | 0 | 0 | 1 | 0 | 1 |
| 0812.5.O | 3 | 5 | 0 | 1 | 0 | 0 |
| 1012.1.B | 3 | 0 | 0 | 2 | 0 | 0 |
| 1012.1.U | 2 | 0 | 0 | 3 | 0 | 0 |
| 1012.1.O | 3 | 1 | 0 | 2 | 1 | 1 |
| 1012.2.B | 1 | 0 | 0 | 1 | 0 | 0 |
| 1012.2.U | 2 | 0 | 0 | 1 | 0 | 0 |
| 1012.2.O | 3 | 0 | 0 | 1 | 0 | 3 |
| 1012.3.B | 0 | 0 | 0 | 0 | 0 | 0 |
| 1012.3.U | 3 | 0 | 0 | 1 | 0 | 1 |
| 1012.3.O | 2 | 0 | 0 | 2 | 0 | 1 |
| 1012.4.B | 2 | 0 | 0 | 1 | 0 | 0 |
| 1012.4.U | 2 | 0 | 0 | 1 | 0 | 1 |
| 1012.4.O | 3 | 0 | 0 | 3 | 0 | 0 |
| 1012.5.B | 1 | 0 | 0 | 0 | 0 | 1 |
| 1012.5.U | 0 | 0 | 0 | 1 | 0 | 0 |
| 1012.5.O | 1 | 0 | 0 | 1 | 0 | 1 |
| 0413.1.B | 0 | 0 | 0 | 0 | 0 | 0 |
| 0413.1.U | 0 | 1 | 0 | 0 | 0 | 0 |
| 0413.1.O | 0 | 0 | 0 | 0 | 0 | 1 |
| 0413.2.B | 0 | 0 | 0 | 0 | 0 | 0 |
| 0413.2.U | 0 | 1 | 0 | 0 | 0 | 0 |
| 0413.2.O | 1 | 0 | 0 | 0 | 0 | 0 |
| 0413.3.B | 0 | 0 | 0 | 0 | 0 | 0 |
| 0413.3.U | 0 | 1 | 0 | 0 | 0 | 0 |
| 0413.3.O | 1 | 0 | 0 | 1 | 0 | 0 |
| 0413.4.B | 0 | 0 | 0 | 0 | 0 | 0 |
| 0413.4.U | 1 | 0 | 0 | 0 | 0 | 0 |
| 0413.4.O | 1 | 1 | 0 | 0 | 0 | 0 |
| 0413.5.B | 1 | 0 | 0 | 0 | 0 | 0 |
| 0413.5.U | 0 | 0 | 0 | 0 | 0 | 0 |
| 0413.5.O | 0 | 1 | 0 | 1 | 0 | 0 |
| 0713.1.B | 4 | 0 | 0 | 0 | 0 | 0 |
| 0713.1.U | 1 | 0 | 0 | 0 | 0 | 0 |
| 0713.1.O | 6 | 0 | 0 | 0 | 0 | 0 |
| 0713.2.B | 0 | 0 | 0 | 0 | 0 | 0 |
| 0713.2.U | 0 | 0 | 0 | 1 | 0 | 0 |
| 0713.2.O | 1 | 0 | 0 | 1 | 0 | 0 |
| 0713.3.B | 1 | 0 | 0 | 0 | 0 | 0 |
| 0713.3.U | 0 | 0 | 0 | 0 | 0 | 0 |
| 0713.3.O | 0 | 1 | 0 | 1 | 0 | 1 |
| 0713.4.B | 0 | 0 | 0 | 0 | 0 | 0 |
| 0713.4.U | 1 | 0 | 0 | 1 | 0 | 0 |
| 0713.4.O | 1 | 0 | 0 | 0 | 0 | 0 |
| 0713.5.B | 1 | 0 | 0 | 0 | 0 | 0 |
| 0713.5.U | 0 | 0 | 0 | 2 | 0 | 0 |
| 0713.5.O | 0 | 0 | 0 | 1 | 0 | 0 |
| 1013.1.B | 1 | 0 | 0 | 0 | 0 | 0 |
| 1013.1.U | 0 | 0 | 0 | 0 | 0 | 0 |
| 1013.1.O | 2 | 1 | 0 | 4 | 0 | 0 |
| 1013.2.B | 3 | 0 | 0 | 0 | 0 | 0 |
| 1013.2.U | 2 | 0 | 0 | 1 | 0 | 0 |
| 1013.2.O | 2 | 0 | 0 | 3 | 0 | 0 |
| 1013.3.B | 0 | 0 | 0 | 0 | 0 | 0 |
| 1013.3.U | 0 | 0 | 0 | 0 | 0 | 0 |
| 1013.3.O | 1 | 2 | 0 | 0 | 0 | 0 |
| 1013.4.B | 0 | 0 | 0 | 0 | 0 | 0 |
| 1013.4.U | 0 | 0 | 0 | 2 | 0 | 0 |
| 1013.4.O | 0 | 0 | 0 | 5 | 0 | 0 |
| 1013.5.B | 0 | 0 | 0 | 0 | 0 | 1 |
| 1013.5.U | 1 | 0 | 0 | 1 | 0 | 0 |
| 1013.5.O | 1 | 0 | 0 | 2 | 0 | 0 |
| 414.1.B | 1 | 0 | 0 | 0 | 0 | 0 |
| 414.1.U | 0 | 0 | 0 | 2 | 0 | 0 |
| 414.1.O | 3 | 0 | 0 | 6 | 0 | 1 |
| 414.2.B | 0 | 0 | 0 | 0 | 0 | 0 |
| 414.2.U | 0 | 1 | 0 | 1 | 0 | 0 |
| 414.2.O | 0 | 0 | 0 | 7 | 0 | 0 |
| 414.3.B | 0 | 0 | 0 | 0 | 0 | 0 |
| 414.3.U | 0 | 0 | 0 | 0 | 0 | 0 |
| 414.3.O | 0 | 0 | 0 | 2 | 0 | 1 |
| 414.4.B | 0 | 0 | 0 | 0 | 0 | 0 |
| 414.4.U | 0 | 1 | 0 | 2 | 0 | 0 |
| 414.4.O | 1 | 3 | 0 | 3 | 0 | 0 |
| 414.5.B | 1 | 0 | 0 | 0 | 0 | 0 |
| 414.5.U | 0 | 0 | 0 | 3 | 0 | 0 |
| 414.5.O | 0 | 1 | 0 | 2 | 0 | 1 |

Species-specific abundance data for Mojave plots. This is the raw data used in R to generate total abundance, species richness, and diversity indices for each plot in each time period. Treatment is represented by B=burned, U=unburned, and O=outside the plots.

| monthyear.block.treatment | *Dipodomys merriami* | *Chaetodipus formosus* | *Neotoma lepida* | *Peromyscus crinitus* | *Onychomys leucogaster* | *Ammospermophilus leucurus* | *Sylvilagus audubonii* |
| --- | --- | --- | --- | --- | --- | --- | --- |
| 0511.1.B | 2 | 1 | 0 | 0 | 0 | 0 | 0 |
| 0511.1.U | 2 | 2 | 1 | 0 | 0 | 0 | 0 |
| 0511.1.O | 9 | 1 | 2 | 0 | 0 | 0 | 1 |
| 0511.2.B | 2 | 0 | 0 | 0 | 1 | 0 | 0 |
| 0511.2.U | 5 | 2 | 0 | 0 | 0 | 0 | 0 |
| 0511.2.O | 1 | 1 | 6 | 0 | 0 | 0 | 0 |
| 0511.3.B | 2 | 0 | 2 | 0 | 0 | 0 | 0 |
| 0511.3.U | 2 | 0 | 1 | 0 | 0 | 0 | 0 |
| 0511.3.O | 5 | 1 | 4 | 0 | 0 | 0 | 0 |
| 0511.4.B | 5 | 1 | 2 | 1 | 0 | 0 | 0 |
| 0511.4.U | 2 | 0 | 0 | 0 | 0 | 0 | 0 |
| 0511.4.O | 1 | 1 | 5 | 1 | 1 | 0 | 1 |
| 0511.5.B | 2 | 1 | 1 | 0 | 1 | 0 | 0 |
| 0511.5.U | 1 | 2 | 1 | 1 | 1 | 0 | 0 |
| 0511.5.O | 3 | 1 | 1 | 0 | 0 | 0 | 0 |
| 0711.1.B | 6 | 2 | 0 | 0 | 0 | 0 | 0 |
| 0711.1.U | 7 | 1 | 1 | 0 | 0 | 0 | 0 |
| 0711.1.O | 3 | 4 | 1 | 0 | 0 | 1 | 0 |
| 0711.2.B | 3 | 1 | 0 | 0 | 0 | 0 | 0 |
| 0711.2.U | 4 | 0 | 0 | 0 | 0 | 0 | 0 |
| 0711.2.O | 2 | 4 | 4 | 0 | 1 | 0 | 0 |
| 0711.3.B | 7 | 1 | 0 | 0 | 0 | 0 | 0 |
| 0711.3.U | 5 | 0 | 0 | 0 | 0 | 0 | 0 |
| 0711.3.O | 5 | 5 | 1 | 0 | 0 | 0 | 0 |
| 0711.4.B | 4 | 0 | 0 | 0 | 0 | 0 | 0 |
| 0711.4.U | 3 | 2 | 0 | 0 | 0 | 0 | 0 |
| 0711.4.O | 3 | 6 | 0 | 0 | 0 | 0 | 0 |
| 0711.5.B | 3 | 0 | 0 | 0 | 0 | 0 | 0 |
| 0711.5.U | 2 | 0 | 2 | 1 | 0 | 0 | 0 |
| 0711.5.O | 6 | 4 | 0 | 1 | 0 | 0 | 0 |
| 1011.1.B | 3 | 0 | 0 | 0 | 0 | 1 | 0 |
| 1011.1.U | 10 | 1 | 0 | 0 | 0 | 0 | 0 |
| 1011.1.O | 13 | 1 | 0 | 0 | 0 | 0 | 0 |
| 1011.2.B | 2 | 1 | 0 | 0 | 0 | 0 | 0 |
| 1011.2.U | 0 | 0 | 0 | 0 | 0 | 0 | 0 |
| 1011.2.O | 10 | 1 | 0 | 0 | 0 | 0 | 0 |
| 1011.3.B | 2 | 0 | 2 | 0 | 0 | 0 | 0 |
| 1011.3.U | 7 | 0 | 0 | 0 | 0 | 0 | 0 |
| 1011.3.O | 12 | 3 | 0 | 0 | 0 | 1 | 0 |
| 1011.4.B | 5 | 0 | 1 | 0 | 2 | 0 | 0 |
| 1011.4.U | 0 | 0 | 0 | 0 | 0 | 0 | 0 |
| 1011.4.O | 7 | 3 | 1 | 0 | 0 | 0 | 0 |
| 1011.5.B | 4 | 0 | 1 | 0 | 0 | 0 | 0 |
| 1011.5.U | 1 | 0 | 0 | 0 | 0 | 0 | 0 |
| 1011.5.O | 9 | 1 | 0 | 0 | 0 | 0 | 0 |
| 0512.1.B | 0 | 0 | 0 | 0 | 0 | 0 | 0 |
| 0512.1.U | 4 | 0 | 0 | 0 | 0 | 0 | 0 |
| 0512.1.O | 11 | 1 | 0 | 0 | 0 | 0 | 0 |
| 0512.2.B | 3 | 1 | 0 | 0 | 0 | 0 | 0 |
| 0512.2.U | 0 | 0 | 1 | 0 | 0 | 0 | 0 |
| 0512.2.O | 6 | 2 | 0 | 0 | 0 | 0 | 0 |
| 0512.3.B | 0 | 0 | 0 | 0 | 0 | 0 | 0 |
| 0512.3.U | 3 | 0 | 0 | 0 | 0 | 0 | 0 |
| 0512.3.O | 7 | 2 | 1 | 0 | 0 | 1 | 0 |
| 0512.4.B | 2 | 1 | 0 | 0 | 0 | 0 | 0 |
| 0512.4.U | 0 | 0 | 0 | 0 | 0 | 0 | 0 |
| 0512.4.O | 6 | 4 | 1 | 0 | 0 | 0 | 1 |
| 0512.5.B | 4 | 1 | 0 | 0 | 0 | 0 | 0 |
| 0512.5.U | 1 | 1 | 0 | 0 | 0 | 0 | 0 |
| 0512.5.O | 5 | 2 | 1 | 0 | 0 | 0 | 0 |
| 0712.1.B | 0 | 0 | 0 | 0 | 0 | 0 | 0 |
| 0712.1.U | 3 | 0 | 0 | 0 | 0 | 0 | 0 |
| 0712.1.O | 4 | 0 | 0 | 0 | 0 | 0 | 0 |
| 0712.2.B | 3 | 0 | 0 | 0 | 0 | 0 | 0 |
| 0712.2.U | 0 | 0 | 0 | 0 | 0 | 0 | 0 |
| 0712.2.O | 6 | 0 | 0 | 0 | 0 | 1 | 0 |
| 0712.3.B | 0 | 0 | 0 | 0 | 0 | 0 | 0 |
| 0712.3.U | 4 | 0 | 0 | 0 | 0 | 0 | 1 |
| 0712.3.O | 5 | 2 | 1 | 0 | 0 | 0 | 0 |
| 0712.4.B | 1 | 1 | 1 | 0 | 0 | 0 | 0 |
| 0712.4.U | 0 | 0 | 0 | 0 | 0 | 0 | 0 |
| 0712.4.O | 4 | 4 | 0 | 0 | 0 | 0 | 0 |
| 0712.5.B | 4 | 1 | 0 | 0 | 0 | 0 | 0 |
| 0712.5.U | 1 | 0 | 0 | 0 | 0 | 0 | 0 |
| 0712.5.O | 7 | 0 | 0 | 0 | 0 | 0 | 0 |
| 1012.1.B | 3 | 0 | 0 | 0 | 0 | 0 | 0 |
| 1012.1.U | 1 | 0 | 0 | 0 | 0 | 0 | 0 |
| 1012.1.O | 3 | 0 | 0 | 0 | 0 | 0 | 0 |
| 1012.2.B | 2 | 0 | 0 | 0 | 0 | 0 | 0 |
| 1012.2.U | 5 | 0 | 0 | 0 | 1 | 0 | 0 |
| 1012.2.O | 6 | 0 | 0 | 0 | 0 | 0 | 0 |
| 1012.3.B | 2 | 0 | 0 | 0 | 0 | 0 | 0 |
| 1012.3.U | 2 | 0 | 0 | 0 | 0 | 0 | 0 |
| 1012.3.O | 4 | 0 | 1 | 0 | 0 | 2 | 0 |
| 1012.4.B | 2 | 0 | 0 | 0 | 0 | 0 | 0 |
| 1012.4.U | 1 | 0 | 0 | 0 | 0 | 0 | 0 |
| 1012.4.O | 3 | 0 | 0 | 0 | 0 | 0 | 0 |
| 1012.5.B | 2 | 0 | 0 | 0 | 0 | 0 | 0 |
| 1012.5.U | 2 | 0 | 0 | 0 | 0 | 0 | 0 |
| 1012.5.O | 5 | 0 | 0 | 0 | 0 | 1 | 0 |
| 0413.1.B | 3 | 0 | 0 | 0 | 0 | 0 | 0 |
| 0413.1.U | 3 | 1 | 1 | 0 | 0 | 0 | 0 |
| 0413.1.O | 7 | 2 | 0 | 0 | 0 | 1 | 0 |
| 0413.2.B | 1 | 0 | 0 | 1 | 0 | 0 | 0 |
| 0413.2.U | 3 | 2 | 0 | 0 | 0 | 0 | 0 |
| 0413.2.O | 8 | 0 | 0 | 0 | 1 | 0 | 1 |
| 0413.3.B | 2 | 2 | 0 | 1 | 0 | 0 | 0 |
| 0413.3.U | 4 | 1 | 0 | 0 | 0 | 0 | 0 |
| 0413.3.O | 6 | 1 | 1 | 1 | 0 | 0 | 0 |
| 0413.4.B | 3 | 1 | 0 | 0 | 0 | 0 | 0 |
| 0413.4.U | 2 | 1 | 0 | 0 | 0 | 0 | 0 |
| 0413.4.O | 7 | 3 | 1 | 3 | 0 | 0 | 0 |
| 0413.5.B | 5 | 0 | 0 | 0 | 0 | 0 | 0 |
| 0413.5.U | 4 | 0 | 0 | 1 | 0 | 0 | 0 |
| 0413.5.O | 7 | 6 | 4 | 2 | 0 | 0 | 0 |
| 0713.1.B | 0 | 0 | 0 | 0 | 0 | 0 | 0 |
| 0713.1.U | 1 | 0 | 1 | 1 | 0 | 0 | 0 |
| 0713.1.O | 3 | 0 | 0 | 1 | 0 | 0 | 0 |
| 0713.2.B | 1 | 1 | 0 | 0 | 0 | 0 | 0 |
| 0713.2.U | 3 | 1 | 0 | 0 | 0 | 0 | 0 |
| 0713.2.O | 7 | 2 | 0 | 0 | 0 | 0 | 0 |
| 0713.3.B | 2 | 0 | 0 | 0 | 0 | 0 | 0 |
| 0713.3.U | 1 | 0 | 0 | 0 | 0 | 0 | 0 |
| 0713.3.O | 5 | 4 | 2 | 0 | 0 | 0 | 0 |
| 0713.4.B | 4 | 0 | 0 | 0 | 0 | 0 | 0 |
| 0713.4.U | 0 | 0 | 0 | 0 | 0 | 0 | 0 |
| 0713.4.O | 3 | 0 | 1 | 0 | 0 | 1 | 0 |
| 0713.5.B | 3 | 0 | 0 | 0 | 0 | 0 | 0 |
| 0713.5.U | 0 | 0 | 0 | 0 | 0 | 0 | 0 |
| 0713.5.O | 2 | 6 | 1 | 1 | 0 | 0 | 0 |
| 1013.1.B | 1 | 0 | 0 | 0 | 0 | 0 | 0 |
| 1013.1.U | 4 | 0 | 0 | 0 | 0 | 0 | 0 |
| 1013.1.O | 4 | 1 | 0 | 0 | 0 | 1 | 0 |
| 1013.2.B | 5 | 0 | 0 | 0 | 0 | 0 | 0 |
| 1013.2.U | 5 | 0 | 0 | 1 | 0 | 0 | 0 |
| 1013.2.O | 3 | 0 | 0 | 0 | 0 | 0 | 0 |
| 1013.3.B | 6 | 0 | 0 | 0 | 0 | 0 | 0 |
| 1013.3.U | 1 | 0 | 0 | 0 | 0 | 0 | 0 |
| 1013.3.O | 5 | 1 | 1 | 1 | 0 | 0 | 0 |
| 1013.4.B | 4 | 0 | 0 | 0 | 0 | 0 | 0 |
| 1013.4.U | 2 | 0 | 0 | 0 | 0 | 0 | 0 |
| 1013.4.O | 6 | 0 | 0 | 0 | 0 | 1 | 0 |
| 1013.5.B | 2 | 0 | 0 | 0 | 0 | 0 | 0 |
| 1013.5.U | 2 | 0 | 0 | 0 | 0 | 0 | 0 |
| 1013.5.O | 3 | 1 | 1 | 0 | 0 | 0 | 0 |
| 414.1.B | 2 | 0 | 0 | 0 | 0 | 0 | 0 |
| 414.1.U | 2 | 0 | 0 | 0 | 0 | 0 | 0 |
| 414.1.O | 3 | 0 | 1 | 0 | 0 | 1 | 0 |
| 414.2.B | 1 | 0 | 0 | 0 | 0 | 0 | 0 |
| 414.2.U | 4 | 0 | 0 | 0 | 0 | 0 | 0 |
| 414.2.O | 4 | 0 | 0 | 0 | 0 | 1 | 0 |
| 414.3.B | 1 | 0 | 0 | 0 | 0 | 0 | 0 |
| 414.3.U | 2 | 0 | 0 | 0 | 0 | 0 | 0 |
| 414.3.O | 5 | 0 | 1 | 0 | 0 | 0 | 0 |
| 414.4.B | 2 | 0 | 0 | 0 | 0 | 0 | 0 |
| 414.4.U | 0 | 0 | 0 | 0 | 0 | 0 | 0 |
| 414.4.O | 5 | 0 | 0 | 0 | 0 | 1 | 1 |
| 414.5.B | 1 | 0 | 0 | 0 | 0 | 0 | 0 |
| 414.5.U | 1 | 0 | 0 | 0 | 0 | 1 | 0 |
| 414.5.O | 3 | 0 | 0 | 0 | 0 | 0 | 0 |
